# Saliva samples as a source of DNA for high throughput genotyping: an acceptable and sufficient means in improvement of risk estimation throughout mammographic diagnostics

**DOI:** 10.1186/s40001-018-0318-9

**Published:** 2018-04-27

**Authors:** U. G. Poehls, C. C. Hack, A. B. Ekici, M. W. Beckmann, P. A. Fasching, M. Ruebner, H. Huebner

**Affiliations:** 1 Women’s Health Center Wuerzburg, Kaiserstrasse 26, 97070 Würzburg, Germany; 2 Department of Gynecology and Obstetrics, University Breast Center Franconia, Comprehensive Cancer Center Erlangen-EMN, University Hospital Erlangen, Friedrich-Alexander University Erlangen-Nuremberg, Universitätsstrasse 21-23, 91054 Erlangen, Germany; 3 Institute of Human Genetics, Friedrich-Alexander-Universität Erlangen-Nürnberg (FAU), Schwabachanlage 10, 91054 Erlangen, Germany

**Keywords:** Breast cancer, Saliva, DNA extraction, DNA quality

## Abstract

**Background:**

Breast cancer screening programs seem to be an insufficient tool for women at high genetic risk for breast cancer. These women are not adequately monitored yet. Genetic testing may improve clearly the quality of breast cancer prevention programs. At present, blood samples are favored for obtaining high-quality DNA; however, DNA can also be obtained by collecting saliva. The aim of this study was, on the one hand, to determine whether saliva sampling is a practicable means to obtain sufficient quantity and quality of DNA and, on the other hand, whether it is accepted by patients throughout mammographic diagnostics.

**Methods:**

67 consecutive women with diagnostic need for mammography with or without a family history for breast cancer were asked for their basic willingness to undergo a genetic testing by saliva sample in addition to standard diagnostics. Saliva samples were analyzed in terms of DNA quantity and quality.

**Results:**

64 (95.6%) women agreed to provide a saliva sample; 3 of them denied participation. And even 63 out of 64 (98.4%) were interested in their specific results. 45 out of 64 samples contained a DNA concentration above 50 ng/µl, 12 samples were between 25 and 50 ng/µl and only 7 of them were under 25 ng/µl with the standard extraction procedure.

**Conclusion:**

A high number of patients seem to accept salvia samples as a risk assessment tool in breast diagnostics and are interested in their specific risk situation. At the same time, it could be demonstrated that it is an effective way to provide high-quality DNA for breast cancer gene analysis. However, it remains to be shown whether it would be possible to integrate it with the same acceptance in a nationwide breast cancer screening program.

**Electronic supplementary material:**

The online version of this article (10.1186/s40001-018-0318-9) contains supplementary material, which is available to authorized users.

## Background

It is well known that breast cancer (BC) incidence increases worldwide. Global Burden of Disease Cancer Collaboration recently reported BC with a total number of 2,422,000 incident cases and 523,000 deaths in women (10,000 in men), making it the leading cause of cancer deaths for women in 2015 [1]. Overall, BC has the highest incidence rate in Germany and is the third leading cause of cancer related death. German national cancer report documents 71,640 incident cases, 17,853 deaths, and a 5-year prevalence of 315,740 in 2013 [2].

Up to 25% of BC may be hereditary [3–5]. It is well known that 20% of hereditary BC is caused by defects in the high penetrance genes *BRCA1* [6], *BRCA2* [7–9], and *TP53* [10–13]. Several intermediate penetrance genes (*CHEK2*, *ATM*, *PALB2*, and *RAD50*) are responsible for about 5% of cases [14–21]. Nonetheless, BC heritability and tumor development based on a genetic defect seem to be more complex than originally expected. Since common lower-risk alleles, of which around 100 have been validated up to the present time through genome-wide association studies, replication and custom genotyping efforts, clarify around 25% of the risk [4, 5, 22]. Currently validated common genetic variations have been summarized in risk prediction models and offer a first possibility to assess the feasibility within early detection and screening programs [23]. Furthermore, first models have been developed to integrate mammographic density, which is an important risk factor for BC [24, 25] and also influences sensitivity and specificity of mammography [26, 27], into risk prediction with genetic variants [28].

The knowledge about genetic BC risk factors will further increase soon. Therefore, the detection of individuals at risk should become much easier. National BC screening programs have not yet taken any genetic risk factors into account. Women at higher risk may be poorly protected due to too long screening intervals until following control and failure to examine by ultrasound and magnetic resonance imaging [29–33]. Women at low risk might receive an unjustified x-ray burden [34].

Genetic testing might offer a suitable way to maximize safety and minimize harm throughout BC screening and could potentially improve the program, and therefore individualize early detection and screening of BC. There is no effort to implement genetic testing in routine mammography so far. This may be due to too high cost or low patient acceptance.

To date, in genetic testing for risk assessment, blood tests are predominantly preferred. Not only the collection of samples is complex and the acceptance could be reduced due to painful blood collection, but there are concrete indications that non-invasive methods are preferred [35].

Sample acquisition using salivary leucocytes are available as an easy method to obtain germline DNA. This might be a chance of introducing genetic testing on a large scale within early detection and screening programs. In this study, we aimed at the analysis of DNA quality for these purposes as well as a first assessment on women’s willingness to take part in genetic assessment for the purpose of individualized early detection and screening programs.

## Methods

### Patients

A total of 67 undergoing a diagnostic mammography necessitated by clinical findings or familial history were asked to participate in our recent study. Three out of 67 denied participation due to unknown reasons. Patients were asked to complete a questionnaire asking basic patient characteristics and their view on individualized early detection and screening of BC (see Additional file 1: Questionaire). Furthermore, they were asked to provide a saliva sample. All patients signed a written informed consent and the study was approved by the Ethics Committee of the Medical Faculty, Friedrich-Alexander University Erlangen Nuremberg (#2100).

### Patient questionnaire

Physical data (age, weight, height and menopausal status), obstetrical data (parity and age at first delivery), personal history per breast biopsy and family history per BC and ovarian cancer (OC) were obtained by questionnaire survey (Additional file 1: Questionaire). All patient questionnaires were anonymized after completion.

### Sampling of saliva and DNA isolation

Saliva from 64 women was collected using Oragene DNA (OG-500) all-in-one system (Genotek, Ottawa, Canada). All samples were completely anonymized. Saliva/OG-500 samples were stored up to 8 weeks at 4 °C until further processing.

For DNA isolation, two different protocols (manual and automated) were used and evaluated considering the complexity of the procedure, DNA yield and DNA quality. Prior to DNA isolation, saliva samples were incubated at 50 °C overnight with gentle agitation in an air incubator for adequate DNA release and permanent nuclease inactivation. The manual purification of DNA was performed using the prepIT L2P (PT-2LP, DNA genotek, Canada) reagent according to the manufacturer’s instructions. In brief, 500 µl of each sample was vortexed with 20 µl of PT-L2P, incubated on ice for 10 min and centrifuged for 5 min at 15,000×*g*. For DNA precipitation, 500 µl of the supernatant was mixed with 600 µl of 95% EtOH. After 10-min incubation at room temperature, samples were centrifuged for 2 min at 15,000×*g*. The DNA pellet was washed with 250 µl of 70% EtOH. Finally, the pellet was dissolved in 80 µl elution buffer. For the automated magnetic bead-based DNA purification, the saliva samples were processed on the Maxwell RSC instrument (Promega, Germany) using the Maxwell RSC Blood DNA Kit. For preprocessing of the samples, 300 µl of saliva was incubated with 30 µl Proteinase K and 300 µl lysis buffer for 20 min at 56 °C. The samples were then added to the cartridges according to the manufacturer’s instructions and automatically processed. DNA was eluted in 80 µl of Maxwell elution buffer.

### Measurement of DNA concentration

The concentration of isolated DNA was analyzed by two different methods. By using the Picodrop Microliter UV/Vis Spectrophotometer, we assessed the concentration and purity of DNA in particular for protein contamination. Additionally, we used the Quantus Fluorometer (Promega, Germany) with the QuantiFluor dsDNA system for a specific quantification of dsDNA. Both measurements were performed according to the manufacturer’s instructions.

### Assessment of DNA quality

The quality of isolated DNA was analyzed by agarose gel electrophoresis [36]. Three microlitres of DNA was loaded onto a 2% agarose gel. Lambda DNA was used as a control as well as a marker for high-quality DNA. Nucleic acids were stained using peqGreen DNA/RNA dye (VWR, Germany). DNA quality was scored depending on molecular weight, grade of degradation, intensity of staining, and RNA contamination as detected by gel electrophoresis, with 1 high, 2 medium, and 3 low quality.

### PCR amplification of bacterial and human DNA

To determine the ratio of human and bacterial DNA content, we performed a PCR amplification using primer sets specific for human beta-globin [37] or bacterial 16s rRNA. Oligonucleotides used for PCR amplification were beta-Globin_TF (sequence CAACTTCATCCACGTTCACC), beta-Globin_BR (sequence GAAGAGCCAAGGACAGGTAC), Ecoli_16s_TF (sequence CCTACGGGAGGCAGCAG), and Ecoli_16s_BR (sequence ATTACCGCGGCTGCTGG). The 16s rRNA gene is not present in human DNA but is conserved across a large range of microorganisms [38]. A total of 200 ng isolated DNA was used per reaction. The regions of interest were amplified using the fast start taq DNA polymerase kit (Sigma-Aldrich) according to the manufacturer’s instructions. To each sample, 0.5 µl of DMSO was added to achieve specific primer binding. For both PCR reactions, an annealing temperature of 55 °C was used. Finally, the amplified fragments were visualized by gel electrophoresis.

### Sequencing of BRCA1 and BRCA2

All three mutations were confirmed in two independent DNAs of each patient. The identity of DNA pairs was confirmed by multiplex PCR with 21 polymorphic markers located on various chromosomes (PowerPlex21, Promega) [39] and analyzed on an automatic capillary sequencer (ABI3500, Applied Biosystems). Both small deletions were confirmed by Sanger sequencing analyzed on an automatic capillary sequencer (ABI3730, Applied Biosystems) as described by Kraus et al. [39]. The three exon deletion was confirmed by multiplex ligation‐dependent probe amplification (MLPA) analysis [40] using the SALSA MLPA kit (BRCA1: P002; MRC Holland) according to the manufacturer’s instructions.

## Results

### Characteristics of participants

A total of 65 out of 67 (97.0%) women could be included into the study. Two women refused to take part. One 74-year-old woman was unable to provide a sample due to xerostomia (dry mouth). Mean age was 53.9 ± 13.3 years. 30 (46.2%) of the respondents were in a premenopausal, 35 (53.8%) in a postmenopausal status. The mean term starting menopause was 48.3 ± 5.9 years. 4 out of 64 (6.3%) participants currently used hormonal replacement therapies. 8 out of 64 (12.5%) women had a history of former breast biopsy, either core or surgical biopsy. 29 out of 65 (44.6%) interviewed persons stated a family history of BC or OC. The average number of affected family members was 1.3 ± 0.6, ranging 1–3. 25 women were from families in which only one female member had BC or OC. 5 women came from families with two affected members. None of the participants stated more than two affected family members. 8 women had affected mothers with BC or OC. One woman indicated that her mother had both, BC and OC, whereby no other family members were affected. All patient characteristics are described in Table 1.

**Table 1:** Characteristics of participants

Characteristics (*n* = 64)	Counts or mean	% or SD
Age	53.9	± 13.3
Parity	1.5	± 1.1
Age at first delivery (years)	25.8	± 4.2
Menopausal status		
Premenopausal	30	46.2%
Postmenopausal	35	53.8%
Age at menopause (years)	48.3	± 5.9
Duration since menopause (years)	15.7	± 9.1
HRT status		
Recent hormonal replacement therapy	4	6.2%
No former hormonal replacement therapy	61	93.8%
Former breast biopsy		
Yes	8	12.3%
No	57	87.7%
Women with at least one affected family member (BC or OC)	29	44.6%
Average number of affected family members	1.3	± 0.6
Women with mothers affected by BC or OC	8	12.3%
Women with mothers affected by BC and OC	1	1.5%
Women with one sister affected by BC	4	6.2%
Women with more than one sister affected by BC	0	0%
Women with affected grandmothers by BC	10	15.4%
Women with at least one other affected relative (e.g., aunts, cousins) by BC	11	16.9%

Characteristics of all women, who participated in the study by giving saliva samples, are listed and quantified by counts (%) or mean ± standard deviation (SD)

With regard to our study aim concerning interest in genetic testing and counseling for individualized early detection or screening, 63 women (98.4% of women who provided a sample, 96.9% of all participants) expressed an interest in genetic analysis and counseling. Only one person said she had no interest.

### Comparison DNA extraction methods

**Fig. 1:**
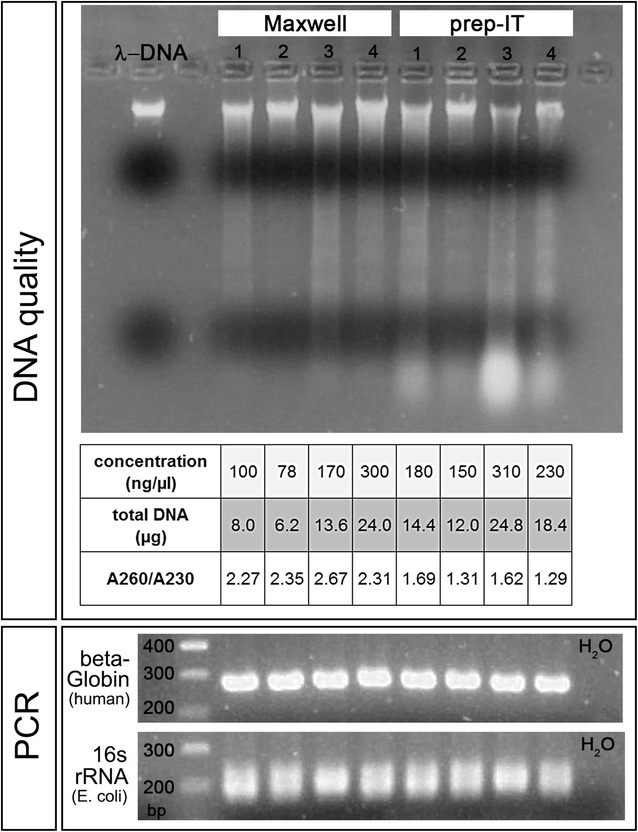
Comparison of quality and bacterial contamination of DNA extracted using two different methods. DNA from four saliva samples (number 1–4) was isolated automatically (Maxwell) or manually (prep-IT). DNA quality was assessed by agarose gel electrophoresis. High-quality DNA is represented by a lambda DNA (300 ng) probe (first lane). DNA quantity was measured by fluorometric assessment (Quantus) and the purity of the DNA was quantified by photometric analysis of absorbance at 260 and 230 nm (A260/A230). PCR amplification of human beta-Globin DNA and bacterial 16s rRNA was used to determine differences in bacterial DNA contamination. In the first lane, a 100 base pairs (bp) DNA marker determines the size of the amplified fragments

In order to determine the quality and quantity of DNA from saliva samples, we isolated DNA from Oragene collection tubes (#1 to 4) using two different methods (Maxwell/automated and prep-IT/manually). We assessed DNA quality by agarose gel electrophoresis. The DNA was of high molecular weight and of similar quantity regardless of the extraction method (Fig. 1). In contrast to that, we observed more signs of degrading and/or shearing of DNA from the prep-IT samples compared to DNA isolated with the Maxwell system (Fig. 1). Additionally, smear at the bottom of the lane indicated contamination of the prep-IT samples with degraded RNA (Fig. 1). Furthermore, purity of DNA was determined by photometric measurement of the absorbance at 260 and 230 nm. In general, expected A260/A230 values are commonly within the range of 2.0–2.2. While we measured a ration above 2.0 for DNA isolated with the Maxwell system, prep-IT DNA exhibited signs of contamination with carbohydrates as the ratio was less than 2.0 (Fig. 1). The amount of DNA differed only slightly between the two extraction methods, with a mean of 13.0 µg total DNA per 300 µl saliva in the Maxwell samples and 17.4 µg DNA per 500 µl saliva in the prep-IT ones (Fig. 1).

Contamination of saliva DNA with bacterial DNA is usual. Thus, we analyzed whether bacterial DNA was present after Maxwell- or prep-IT DNA extraction. We amplified human beta-Globin DNA and 16s ribosomal RNA (rRNA) from *Escherichia coli* by PCR and analyzed the amount by agarose gel electrophoresis. We did not observe any differences between the DNA samples regarding the content of beta-Globin and 16s rRNA (Fig. 1).

### DNA quality

**Fig. 2:**
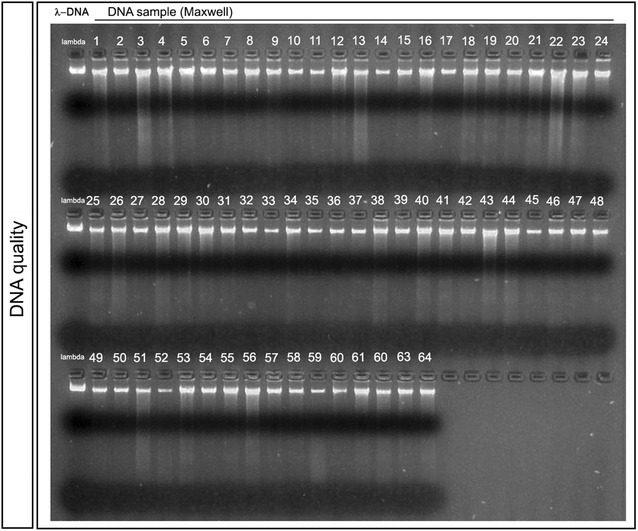
DNA quality assessment by agarose gel electrophoresis. The quality of extracted DNA was analyzed by agarose gel electrophoresis. Three microlitres of each DNA sample (number 1–64) was loaded onto a 2% agarose gel. The first lane shows lambda DNA (300 ng), which was used as a high-quality control, followed by the isolated DNA samples

Since DNA isolated with the Maxwell system showed an overall better purity and quality with similar yield, we used this method to extract DNA from all 64 saliva samples. We determined the DNA quality of the 64 DNA probes using agarose gel electrophoresis. A tight band with a molecular weight similar to lambda DNA (48 kbp) could be visualized for all analyzed probes. Twenty-five samples showed a tight band with no detectable smearing representing highest DNA quality. For 25 additional DNA probes, we found minimal smearing within the higher molecular weight range. 14 of the 64 samples showed degraded DNA reaching to the bottom of the lane, but still with the largest proportion of DNA at a molecular weight of about 48 kbp (Fig. 2).

### DNA concentration

**Fig. 3:**
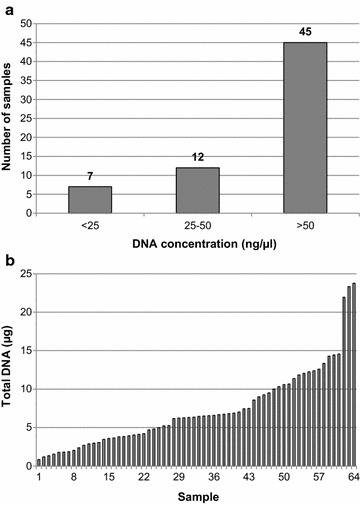
Concentration of DNA. DNA concentrations of all 64 samples were determined by fluorometric measurement (Quantus). Samples were assembled into three groups of low (less than 25 ng/µl), medium (between 25 and 50 ng/µl), and high (more than 50 ng/µl) DNA concentrations (**a**). The total DNA content of each sample was calculated and visualized in a waterfall diagram (**b**)

The DNA concentration was measured using the Quantus Fluorometer. We divided the DNA samples into three different groups depending on the DNA concentration requested for consortium-based genotyping or sequencing arrays e.g., OncoArray (required > 50 ng/µl; in exceptional cases possible 25–50 ng/µl; too low < 25 ng/µl). 7 of 64 probes had concentrations of less than 25 ng/µl, and 12 had a medium concentration between 25 and 50 ng/µl (Fig. 3a). The largest proportion of DNA samples (70%) had a concentration of more than 50 ng/µl (Fig. 3a). A total DNA amount above 5 µg could be detected within 61% of all isolated DNA probes (Fig. 3b). The mean amount of DNA was 7.32 µg per 300 µl saliva.

### Detection of BRCA1 and BRCA2 mutations

In order to compare DNA isolated from blood draws and DNA from saliva in regard to the detection of germline mutations, we sequenced blood and saliva DNA from three different individuals with known mutations in the BRCA1 or BRCA2 loci. For all three individuals, we detected the identical BRCA1 and BRCA2 mutations in DNA from saliva and from blood. Observed mutations, gene, and source of DNA are listed in Table 2. We further confirmed that DNA from blood draws and from saliva collection belonged to the same individual by microsatellite analysis (data not shown).

**Table 2:** Sequencing of BRCA1 and BRCA2

Individual	Gene	Mutation	Source of DNA
01	BRCA1	Deletion of Exons 1a, 1b and 2, IARC class 5. HGVS:c.(?_-200)_(80+1_81-1)del	Blood
01	BRCA1	Deletion of Exons 1a, 1b and 2, IARC class 5. HGVS:c.(?_-200)_(80+1_81-1)del	Saliva
02	BRCA2	Exon 11:c.6379delA heterozygote; p.(Ser2127Valfs*10): IARC class 5	Blood
02	BRCA2	Exon 11:c.6379delA heterozygote; p.(Ser2127Valfs*10): IARC class 5	Saliva
03	BRCA1	Exon 2: c.68_69delAG heterozygote, p./Glu23Valfs*17) (BIX: 18delAG); IARC class 5 (pathogen)	Blood
03	BRCA1	Exon 2: c.68_69delAG heterozygote, p./Glu23Valfs*17) (BIX: 18delAG); IARC class 5 (pathogen)	Saliva

## Discussion

Nationwide Screening programs are established in 19 out of 28 European countries. Finland was the first to be started in 1989. The latest, however, were Spain, Latvia, Germany and Malta in 2009, and Denmark in 2010 [41]. Although most of the programs are population-based, up to now none of them are risk-based. Hence, nowadays national BC screening programs are non-personalized means in cancer detection and individual risks are not taken into any account. Nevertheless, percent mammographic breast density (PMD), structural features, familial history, and genetic variants are very well known as serious risk factors. Regarding the knowledge of several risk factors, there is a strong need for an individualized screening approach.

PMD is a well-known, strong risk factor with evidence of a genetic basis [42]. Ultimately, the entire number of genetic variants increasing BC risk is unclear. Hence, there is evidence suggesting that the presence of varying SNPs correlated with increased PMD and BC risk. Because there seems to be a strong connection between genetic characteristics and mammographic patterns, the question should be, whether an individualized BC detection approach should mention only density, genetic parameters or both. Lee et al. recently showed advantage of BC detection rate in a statistical model using both entities in an Asian population [43].

History of genetic testing in BC risk assessment goes back to the early 1990s [44]. Unfortunately, from that time until present, it is limited to a group of women with extended family history. Expanded BC screening is only offered to women with a lifetime risk above 20% based on estimates [23]. By extending and reinforcing of genetic testing beyond, e.g., BRCA1/2, the number of women who are candidates for extensive screening (like breast magnetic resonance imaging) could be significantly increased [45]. Most of those would not have been identified through family history assessment. On the other hand, there may be a part of population which is over-protected by nationwide screening, because they are at extremely low risk. To detect these individuals and remove them from the screening program or modify the program by creating a low-risk group may decrease potential harm by unnecessary X-ray examinations.

Little scientific information exists about willingness of genetic testing in cancer. There are few data about readiness for genetic testing in prostatic cancer. For example, 74% of prostatic cancer patients are probably or definitely interested in genetic analysis [46]. BC patients’ willingness to accept genetic testing seems to be similarly and reaches 74.1% in a Chinese population [47]. Nevertheless, there are no data available about acceptance of genetic testing in a preventive setting. Results of a previous study [48] indicate that there is a correlation between knowledge about risk and willingness to join diagnostic or even therapeutic prevention programs. So, one way to achieve a high acceptance of genetic testing could be to facilitate a sufficient educational program. The other could be offering a special method of genetic testing with acceptance as high as possible amongst patients and screening participants, respectively.

In general, acceptance rates of genetic testing vary from 20 to 90% [49–51]. When analyzing the acceptance of genetic testing in subject to arising expenses, it was shown that there was a decreasing willingness with rising costs for patients [52–54]. Additionally, Adamkova et al. [35] showed a readiness for genetic testing in a group of healthy study volunteers depending on the sampling method used. The acceptance of blood sampling (72%) and buccal swab sampling (98%) differed considerable and consequently could be substantially improved by using the right method. These data are totally in line with our acceptance findings. We showed that in a routine early detection almost all women (98%) are interested in learning more about individualized early detection including genetic testing.

Because buccal swab and, as we showed, saliva sampling are extremely high accepted methods of genetic testing in healthy women presented for early detection of BC, we were interested whether quality and quantity of DNA from saliva samples could be adequate for testing of genetic risk factors. In that sense, we could extract a suitable quantity of DNA from saliva samples. The amount was comparable to other studies. For example, Looi et al. [55] extracted an average of 15 µg DNA from 1.0 ml saliva, while we observed a mean DNA yield of 7.3 µg from 0.3 ml saliva. When comparing two methods for isolation of DNA from saliva samples, we found that the automated, magnetic bead-based Maxwell RSC system was superior to manual purification with the prepIT L2P within our small test cohort. In particular, the purity of the isolated DNA was better using the Maxwell RSC system. Nevertheless, the bacterial content was identical and thus for both methods a specific quantification of human DNA (e.g., real-time PCR) would be necessary to determine the exact amount [56, 57]. In earlier studies, it was shown that the mean number of bacterial cells per milliliter saliva is approximately 1.7 × 10^7^, which represents about 90% of the total extracted DNA [58]. On the other hand, regardless of the amount of non-human DNA, it was shown that results obtained from further downstream studies, like sequencing or genotyping, were definite and accurate [56, 59]. Similar results could be obtained by us. Using Sanger sequencing or Multiplex ligation-dependent probe amplification, we detected identical mutations within BRCA1 and BRCA2 in DNA from blood and saliva. Taken together, we were able to show well suitable extraction methods for the isolation of DNA from saliva with high quality and quantity, a high interest in genetic testing when using saliva as a DNA source and a concordance between sequencing results of blood-based and saliva-based DNA. In summary, this makes saliva a well-suited source for a variety of studies and especially for the detection of cancer biomarkers (e.g., specific germline mutations in BRCA1 and BRCA2) during screening procedures [60].

## Conclusion

Saliva sampling seems to be sufficient means for high throughput genotyping used for BC-risk stratification. In addition, it is accepted by patients in a diagnostic mammography setting due to its simple application. This might help to detect individuals at high risk who have need for intense surveillance. On the other side, it might be possible to detect women at low risk to avoid needless x-ray exposure in the sense of longer screening intervals. Further investigation is needed to prove applicability in BC screening program.

## Additional file

**Additional file 1: Questionaire.** Physical data (age, weight, height and menopausal status), obstetrical data (parity and age at first delivery), personal history per breast biopsy and family history per BC and ovarian cancer (OC) were obtained by questionnaire survey

## Abbreviations

BC: breast cancer
OC: ovarian cancer
bp: base pairs
rRNA: ribosomal RNA
PMD: percent mammographic breast density
TF: top forward
BR: bottom reverse
SNP: single-nucleotide polymorphism
