# Supplementary material for: Saliva samples as a source of DNA for high throughput genotyping: an acceptable and sufficient means in improvement of risk estimation throughout mammographic diagnostics
Source: Eur J Med Res. 2018 Apr 27;23:20. doi: 10.1186/s40001-018-0318-9 (PMC5921411; doi:10.1186/s40001-018-0318-9)
Supplement: Supplementary file 1 — Additional file 1: Questionaire. Physical data (age, weight, height and menopausal status), obstetrical data (parity and age at first delivery), personal history per breast biopsy and family history per BC and ovarian cancer (OC) were obtained by questionnaire survey [file 40001_2018_318_MOESM1_ESM.pdf]

► What is your height? ………..… cm	► What is your weight? …….…….kg
► What is your date of birth? ………..…	► How old are you? ……..……years
► Do you have a regular menstrual period?<br>► if not, since when?	☐ no ☐ yes<br>Since month:….... year:………
► Do you use hormonal replacement therapy?	☐ no ☐ yes
► How many children do you have?<br>► What was your age at your first delivery?	………children<br>at the age of ……..years
► Did you ever underwent a breast biopsy? (e.g. surgery or needle biopsy)	☐ no ☐ yes
► Did your mother have a history of breast cancer?<br>► if yes, at which age?	☐ no ☐ yes<br>at the age of ……..years
► Did your mother have a history of ovarian cancer?<br>► if yes, at which age?	☐ no ☐ yes<br>at the age of ……..years
► Do you have sisters?<br>► Did one or more of your sister have a history of breast cancer?<br>► If yes, at which age?<br>sister 1:<br>sister 2:	☐ no ☐ yes<br>☐ no ☐ yes<br><br>at the age of ……..years<br>at the age of ……..years
► Did one or more of your sister have a history of ovarian cancer?<br>► If yes, at which age?<br>sister 1:<br>sister 2:	☐ no ☐ yes<br><br>at the age of ……..years<br>at the age of ……..years
► Are there any other family members with a history of breast cancer?	☐ no ☐ yes
► if yes, who? **person 1** ……….. at the age of …years **person 2**…………..… at the age of ……..years	
► Are there any other family members with a history of ovarian cancer?	☐ no ☐ yes
► if yes, who? **person 1** ……….. at the age of …years **person 2**…………..… at the age of ……..years	
► If analyzed, would you like to know the genetic result and corresponding risk?	☐ no ☐ yes
